# Prediction of pure tone thresholds using the speech reception threshold and age in elderly individuals with hearing loss

**DOI:** 10.1186/s13104-024-06762-3

**Published:** 2024-04-17

**Authors:** Ramida Dindamrongkul, Tippawan Liabsuetrakul, Pittayapon Pitathawatchai

**Affiliations:** 1 Department of Otolaryngology, Faculty of Medicine, Prince of Songkla University, 90110 Hatyai, Songkhla Thailand; 2 Epidemiology Unit, Faculty of Medicine, Prince of Songkla University, Hatyai, Songkhla Thailand

**Keywords:** Elderly, Predictive model, Pure tone threshold, Speech reception threshold

## Abstract

**Objective:**

Early detection and effective management of hearing loss constitute the key to improving the quality of life of individuals with hearing loss. However, in standardized pure tone audiometry, it is sometimes difficult for elderly patients to understand and follow all instructions. Audiologists also require time, expertise, and patience to ensure that an elderly can identify the faintest levels of stimuli during a hearing test. Therefore, this study aimed to devise and validate a formula to predict the pure tone threshold at each frequency across 0.5–4 kHz (PTTs) using speech reception threshold.

**Methods:**

The 1226 audiograms of hearing-impaired individuals aged 60–90 years were reviewed. The random sample function randomly assigned 613 participants to the training and testing sets each. A linear model was created to predict the PTT value at each frequency based on variables significant at all frequencies across 0.5–4 kHz. The adjusted-R2 value was considered to indicate the performance of the predictive model. Pearson’s correlation coefficient was used to describe the relationship between the actual and predicted PTT at 0.5, 1, 2, and 4 kHz among the testing set to measure the performance of the proposed model.

**Results:**

The predictive model was devised using variables based on the speech recognition threshold (SRT) after adjusting with age in the training set. The overall prediction accuracy demonstrated a higher adjusted-R^2^ ranging from 0.74 to 0.89 at frequencies of 0.5, 1, and 2 kHz, whereas a low percentage of explained variance was observed at 4 kHz (adjusted-R^2^ = 0.41). This predictive model can serve as an adjunctive clinical tool for guiding determination of the PTTs. Moreover, the predicted PTTs can be applied in the hearing aid programming software to set appropriate hearing aid gain using standard prescriptive formulas.

## Introduction

Sensorineural hearing loss is a common sensory deterioration that generally occurs in the elderly, with a high global prevalence. Pure-tone audiometry is the gold standard audiologic examination to assess hearing acuity and ability [1–3]. The severity of hearing loss is ascertained using the pure-tone average (PTA) obtained from four pure-tone thresholds (PTTs) at frequencies of 0.5, 1, 2, and 4 kHz [4]. Early hearing aid fitting should be performed to enhance sound amplification and decrease the listening effort due to hearing difficulties. Hearing aids also enhance hearing ability and improve social engagement, which are related to the patient’s quality of life and health satisfaction [5, 6]. Although hearing aids provide various benefits, patients seldom seek them, possibly due to insufficient access to hearing evaluations. In low- and middle-income countries, over 85% of individuals who need hearing aids lack access to the same [7]. In southern Thailand, it was revealed that nearly 50% of individuals with hearing disabilities lived more than 100 km away from a main audiology and hearing aid center, Songklanagarind Hospital, which required at least 2 h of driving [8].

The respective PTTs across the frequencies 0.25 to 8 kHz are required to input hearing levels into a hearing aid programming software to set the necessary gain based on the selected prescriptive formula. However, determining the PTTs is challenging in elderly individuals, owing to various physical and psychological factors including frailty, anxiety, delirium, reduced coordination, dizziness, muscle weakness, loss of cognitive function, and sensory changes [9–11]. Furthermore, elderly individuals may provide slow, hesitant, and difficult responses to unfamiliar sound stimuli during hearing investigation [2, 12–14]. Standardized pure-tone audiometry for the elderly is a time-consuming process that requires expertise, and patience on the part of an audiologist to ensure the identification of the faintest levels of stimuli at different pitches [13, 15–17]. Inaccurate and inconsistent PTTs could be responsible for improper amplification in hearing aid fitting, which may adversely affect elderly individuals with disability [18–21]. In common practice when pure tone audiometry cannot be completed, speech audiometry is recommended as an alternative for audiologists to obtain speech recognition thresholds which can agree closely to PTA [22]. The benefits to assess hearing thresholds with a speech stimulus include a validity check for pure tone audiograms, assessment of non-organic hearing impairment and a hearing aid evaluation [23].

Early detection of hearing loss followed by optimal intervention, especially hearing aid fitting, is important for ensure a good quality of life for the elderly [5, 6, 24, 25]. The hearing-aid fitting procedure is intended to emphasize speech communication; hence, a speech stimulus is key to evaluating the hearing level, providing an overview of speech intelligibility in the recognition and listening tasks. The speech recognition threshold (SRT) is a speech-based threshold that provides more realistic daily listening that is obtained with the PTT during standard audiometry [26–28]. Most studies have shown a good correlation between the SRT and PTA [26, 29–33] and the former is routinely used clinically to validate the reliability of audiometry. However, the SRT is not related to the PTT at each specific frequency. In terms of acoustic parameters, a pure tone stimulus is a single specific frequency, whereas a speech stimulus consists of a broad spectrum of frequencies and includes both vowels and consonants. Thus, the speech stimulus conveys multiple acoustic cues from vowels and intonation which is easier to be recognized compared to the pure tone stimulus [28].

Age was not only related with the peripheral auditory system, but also involved the central auditory processing which could affect speech comprehension [34]. Regarding cognitive decline and hearing loss, the difficulty in speech recognition was obviously seen in a noise condition. Any noise condition could deprive speech intelligibility resulting in poor SRT due to altered temporal processing in elderly individuals [35]. Age-related changes in physical and cognitive function in elderly individuals could lead to inconsistent and inaccurate audiometric assessment [36–38]; thus, the acquisition of PTTs across frequencies of 0.5–4 kHz with optimal consistency and accuracy in elderly individuals is a challenging endeavor. Therefore, this study aimed to determine which parameters among audiometric and demographic data are the significant predictors and should be used to create the predicted formulas for estimating PTTs among older adults. The predictive model can be an adjunctive clinical tool to guide audiologists in any setting where complete PTTs in elderly individuals are difficult to obtain. Moreover, the efficiency of hearing examination in lack of healthcare providers can be enhanced when this predictive model is used. Also, an initial threshold baseline for the prescription of hearing aid amplification can be acquired based on the predictive model.

## Materials and methods

This retrospective study was conducted at a tertiary hospital in southern Thailand between January and June 2022. The study protocol was approved by the Human Research Ethics Committee of the Faculty of Medicine, Prince of Songkla University and conducted in compliance with the Declaration of Helsinki. The data of participants aged 60–90 years who were diagnosed with hearing loss between January 2011 and December 2021 were reviewed. The diagnosis of hearing loss was based on the International Classification of Diseases and Related Health Problems, 10th version, using codes H903, H904, and H905 for sensorineural hearing loss. Audiograms with PTA exceeding 80 dB HL were excluded. In addition, audiograms with SRT-PTA discrepancy over than 12 dB HL suggesting nonorganic hearing loss were excluded [39]. Pure tone and speech audiometry were retrieved on the better ear which was performed at the same time in soundproof room. Although no clear evidence showed the differences in the relationship of SRT and PTT between ears in the literature, only the better ear was selected for data inclusion in this study. Using the better ear could provide more sample size from our database as the worse ear tended to show PTA exceeding 80 dB HL and needed to be subsequently excluded. Regarding a speech material used, RAMA-SRT1 is a Thai-tonal disyllabic words with equal loudness which was applied with a live-voice presentation [40].

The data of eligible participants were retrieved from the Hospital Information System and the Division of Digital Innovation and Data Analysis. After extraction, the medical records of eligible participants were reviewed to determine if they met the inclusion. The inclusion criteria were elderly with slight, moderate, or severe sensorineural hearing loss on the better ear, aged over 60 to 90 years on the date of hearing examination. The hearing ability from 1758 audiograms was classified as slight, moderate, or severe hearing loss. Thereafter, 532 audiograms were excluded because they were incomplete (147, 8.36%) or the PTA-SRT discrepancies exceeded 12 dB HL (385, 21.89%). Finally, 1226 audiograms were included and randomly divided into the training and testing sets using the random sample function command in R, which assigned 613 participants to the training and testing sets each. The training set was used for creating the predictive formulas, whereas the testing set was then applied to assess the correlation between actual and predicted PTTs.

The data were generated with an algorithm using the “set.seed(value)” function to create random objects based on a sequence of generated values. Subsequently, the data were subjected to random sampling, which was grounded on the parameters provided in the function call by the “sample(data, n)” function. The Wilcoxon signed-rank and Pearson’s chi-squared tests were used to compare the baseline characteristics of participants in the training and testing sets to verify that the datasets were not different. Also, a paired t-test statistical procedure was used to determine the discrepancies between (i) SRT and PTA and (ii) SRT and PTTs at different frequencies.

The main outcome measure in this study was the PTT at each frequency. The data of all participants were reviewed for the SRT results, age, sex, hearing aid fitting, and related symptoms, including cognitive loss, tinnitus, and movement disorders. Comorbidities such as diabetes mellitus, cerebrovascular diseases, hypertensive diseases, disorders of lipoprotein metabolism, and depressive episodes, were also recorded. The SRT and PTT were evaluated in the same session. The PTA was calculated from the PTT at 0.5, 1, 2 and 4 kHz in the better ear.

Univariate linear regression was performed for each PTT and variable in the training set data. The “ lm( )” function, which is used to fit linear models in R, was applied to statistically significant variables. A linear model was created to predict the PTT value at each frequency based on variables significant at all frequencies across 0.5–4 kHz. The adjusted-R^2^ value was considered to indicate the performance of the predictive model. Finally, Pearson’s correlation coefficient was used to describe the relationship between the actual and predicted PTT at 0.5, 1, 2, and 4 kHz among the testing set to measure the performance of the proposed model.

Data were analyzed using R software, version 3.4.0 (R Foundation, Vienna, Austria). In the training set, the association between the SRT and PTT was analyzed via correlation analysis using Pearson’s correlation coefficient, as appropriate. Multiple regression analysis was performed to determine the predictive relationship between PTT(y) and SRT (x) with other variables, and to assess the performance using the adjusted-R^2^. In the testing set, calibration of the PTT predicted by the model and the actual PTT was analyzed for internal validation of the model using Pearson’s correlation coefficient. A two-tailed *p*-value less than 0.05 was considered to be statistically significant.

## Results

The baseline characteristics of the participants in the training and testing sets did not show significant differences (Table 1). Audiometric and demographic data of 1226 participants were reviewed. Moderate hearing loss was observed in 40% of participants, whereas slight and severe loss occurred in 37.2% and 22.8% of participants, respectively. Comorbidities, such as hypertension and diabetes mellitus were present in almost 50% of patients in the training and testing sets. There were no significant differences in SRT, PTA, or individual PTT between the participants assigned into the training set and the testing set (Table 2). The mean SRT for the training and testing sets was 44.2 (SD = 16.0) and 43.5 (SD = 15.8), respectively. The mean PTTs at 0.5, 1, 2, and 4 kHz ranged from 39.8 dB HL to 59.0 dB HL.

**Table 1:** Baseline characteristics of the participants

Characteristic	Total (*n* = 1226) no. (%)	Patients Training Set (*n* = 613) no. (%)	Patients Testing Set (*n* = 613) no. (%)	*P*-value
Age Group (years)				
60–69	446 (36.4)	225 (36.7)	221 (36.1)	0.919
70–79	494 (40.3)	248 (40.5)	246 (401)	
80–89	286 (23.3)	140 (22.8)	146 (23.8)	
Gender				
Female	634 (51.7)	315 (51.4)	319 (52)	0.819
Male	592 (48.3)	298 (48.6)	294 (48)	
Severity of hearing loss				
Slight hearing loss (26–40 dB HL)	449 (36.6)	221 (36.1)	228 (37.2)	0.819
Moderate hearing loss (41–60 dB HL)	485 (39.6)	240 (39.2)	245 (40.0)	
Severe hearing loss (61–80 dB HL)	292 (23.8)	152 (24.8)	140 (22.8)	
Related symptoms				
Tinnitus	431 (35.2)	226 (36.9)	205 (33.4)	0.209
Cognitive impairment	358 (29.2)	173 (28.2)	185 (30.2)	0.451
Movement disorders	350 (28.5)	162 (26.4)	188 (30.7)	0.100
Underlying diseases				
Diabetes mellitus	691 (48.2)	301 (49.1)	390 (47.3)	0.530
Cerebrovascular diseases	248 (20.2)	120 (19.6)	128 (20.9)	0.570
Hypertension	607 (49.5)	298 (48.6)	309 (50.4)	0.530
Disorders of lipoprotein metabolism	485 (39.6)	253 (41.3)	232 (37.8)	0.220
Depressive episode	52 (4.2)	31 (5.1)	21 (3.4)	0.156
Hearing aid fitting	241 (19.7)	119 (19.4)	122 (19.9)	0.829

Values are presented as number and percentage

**Table 2:** Comparison mean and standard deviations of training set and testing set in speech reception threshold (SRT), pure-tone average (PTA), pure-tone threshold (PTT) at 0.5, 1, 2, and 4 kHz

	Patients Training Set (*n* = 613) Mean ± SD	Patients Testing Set (*n* = 613) Mean ± SD	*P*-value
SRT	44.2 ± 16.0	43.5 ± 5.8	0.471
PTA	48.2 ± 14.7	47.8 ± 4.4	0.658
PTT at 0.5 kHz	40.9 ± 16.2	39.8 ± 15.7	0.247
PTT at 1 kHz	44.4 ± 16.0	43.7 ± 16.5	0.405
PTT at 2 kHz	48.6 ± 16.9	48.8 ± 16.6	0.779
PTT at 4 kHz	58.9 ± 17.3	58.9 ± 16.7	0.955

Values are presented as mean ± standard deviation

The discrepancies among the SRT, the PTT and PTA at different frequencies in the training set (*n* = 613 participants) are shown in Table 3. The hearing levels increased continuously and reached the maximum value at a PTT of 4 kHz, with the increase in the frequency of the pure-tone stimuli. The discrepancies in hearing levels differed significantly between the SRT and PTA and all limits of the PTT, except at 1 kHz. The highest discrepancy of the hearing levels between SRT and PTT was found in the PTT at 4 kHz and was the lowest at 1 kHz. Generally, the SRT was lower than the pure-tone hearing threshold at each frequency, except the PTT at 0.5 kHz. Furthermore, Table 3 also demonstrated the discrepancies between actual PTTs and predicted PTTs at different frequencies, which were lower than 1 dB HL.

**Table 3:** Threshold discrepancy among pure-tone threshold (PTT), pure-tone average (PTA), speech reception threshold (SRT) and predicted pure-tone threshold (PTT) in training set

	Hearing threshold (dB HL)			Discrepancy of hearing level (dB HL)							
				Pure tone levels - SRT				Actual PTT - Predicted PTT			
	Mean		SD	Mean		SD	*p*-value	Mean		SD	*p*-value
SRT	44.2		16.0								
PTA	48.2		14.7	4.0		5.5	< 0.001*				
PTT at 0.5 kHz	40.9		16.2	-3.3		7.0	< 0.001*	0.03		6.8	0.576
PTT at 1 kHz	44.5		16.0	0.3		5.5	0.752	0.02		5.4	0.843
PTT at 2 kHz	48.6		16.9	4.3		8.7	< 0.001*	0.2		8.6	0.965
PTT at 4 kHz	59.0		17.3	14.7		14.2	< 0.001*	-0.4		13.3	0.653

*P < 0.05

**Fig. 1:**
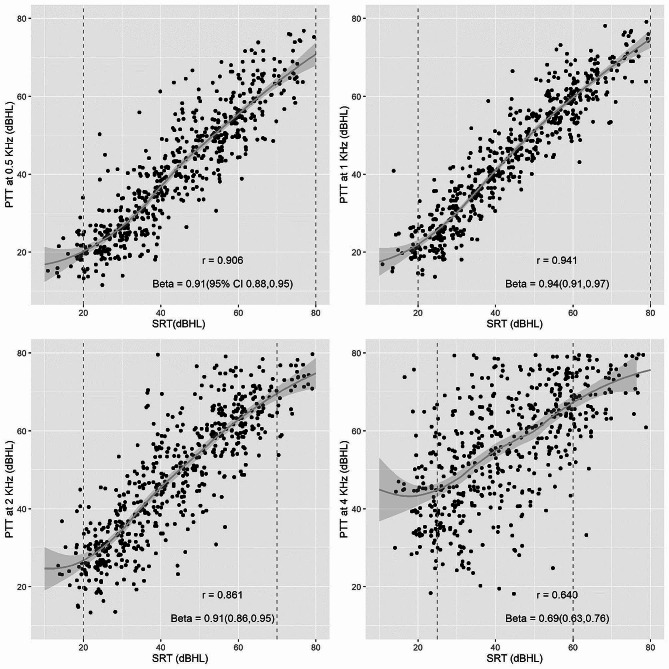
Pearson’s correlation coefficient describing the relationship between the speech reception threshold (SRT) and pure-tone threshold (PTT) at 0.5, 1, 2, and 4 kHz among training set

The correlations between SRT and PTTs at each threshold are presented in Fig. 1. Moderate-to-robust correlations were observed between hearing levels measured by SRT and PTT. The results of univariate linear regression for each PTT by SRT and other factors are shown in Table 4. SRT, age, tinnitus, and hypertension were significant predictors of the PTT at frequencies of 1 and 2 kHz, while the SRT, age, tinnitus and cerebrovascular disease were significant predictors at 0.5 kHz. Only SRT and age were significant predictors of the PTT at 4 kHz.

**Table 4:** Univariate linear regression of each pure tone thresholds (PTT) with various variables

Variables	PTT at 0.5 kHz		PTT at 1 kHz		PTT at 2 kHz		PTT at 4 kHz	
	β (95% CI)	*p*-value	β (95% CI)	*p*-value	β (95% CI)	*p*-value	β (95% CI)	*p*-value
Speech reception threshold	0.91 (0.88,0.95)	< 0.001*	0.94 (0.91,0.97)	< 0.001*	0.91 (0.86,0.95)	< 0.001*	0.69 (0.63,0.76)	< 0.001*
Sex	-0.85 (-3.41,1.72)	0.5173	-1.2 (-3.75,1.34)	0.3525	-0.41 (-3.09,2.27)	0.7661	-0.42 (-3.17,2.34)	0.7662
Age	0.36 (0.19,0.53)	< 0.001*	0.42 (0.25,0.59)	< 0.001*	0.47 (0.29,0.64)	< 0.001*	0.45 (0.27,0.63)	< 0.001*
Tinnitus	-4.68 (-7.32,-2.05)	< 0.001*	-4.78 (-7.39,-2.17)	< 0.001*	-4.07 (-6.83,-1.31)	< 0.001*	-2.73 (-5.57,0.12)	0.06022
Cognitive impairment	-0.6 (-3.45,2.25)	0.6798	-1.17 (-3.99,1.66)	0.418	-0.7 (-3.67,2.28)	0.6454	-1.2 (-4.26,1.85)	0.4398
Movement disorders	-0.62 (-3.53,2.29)	0.6741	0.33 (-2.55,3.22)	0.8212	0.94 (-2.09,3.98)	0.5418	0.66 (-2.46,3.78)	0.6762
Diabetes mellitus	-0.37 (-2.94,2.19)	0.7744	-0.57 (-3.12,1.97)	0.6579	-0.28 (-2.96,2.4)	0.8375	-0.92 (-3.67,1.83)	0.5123
Cerebrovascular diseases	3.46 (0.24,6.69)	0.0351*	2.46 (-0.74,5.66)	0.1311	1.99 (-1.38,5.37)	0.2466	0.73 (-2.74,4.2)	0.6802
Hypertension	-1.73 (-4.29,0.84)	0.1861	-3.52 (-6.05,-0.99)	0.0064*	-2.95 (-5.62,-0.28)	0.0303*	-1.95 (-4.7,0.8)	0.1638
Disorders of lipoprotein metabolism	-1.64 (-4.24,0.97)	0.2175	-2.07 (-4.65,0.5)	0.1148	-2.47 (-5.18,0.25)	0.07462	-1.45 (-4.25,1.34)	0.3074
Depressive episode	0.05 (-5.81,5.91)	0.987	0.21 (-5.59,6.01)	0.9433	2.19 (-3.92,8.3)	0.4817	5.35 (-0.92,11.61)	0.09432
Hearing aid fitting	1.98 (-1.26,5.23)	0.2296	1.12 (-2.1,4.33)	0.4951	0.9 (-2.49,4.28)	0.604	2.34 (-1.14,5.81)	0.1868

Values are presented as Beta (β) and 95% confidence intervals

*P < 0.05

The resultant multiple regression model is presented in Table 5. This model was used to analyze PTT prediction at 0.5, 1, 2, and 4 kHz. The resultant multiple regression model for PTT prediction was formulated using the variables in training set significant at all frequencies across 0.5–4 kHz. Even though the predictors including SRT, age, hypertension, tinnitus and cerebrovascular diseases were significant predictors among some specific frequencies in 0.5–4 kHz. Only two, SRT and age, were significant predictors across all frequencies of predicted PTTs from 0.5 to 4 kHz. Then, both SRT and age were calculated for a higher value of adjusted-R^2^ indicated a significantly better performance of the model for predicting the PTT at 1 kHz (adjusted-R^2^ = 0.89) and 0.5 kHz (adjusted-R^2^ = 0.82), followed by 2 kHz (adjusted-R^2^ = 0.74) and 4 kHz (adjusted-R^2^ = 0.41), respectively.

**Table 5:** The prediction model of pure-tone threshold (PTT) at 0.5, 1, 2, and 4 kHz

Predicted PTT	Resultant multiple regression model	Adjusted-R^2^	Prediction model	Adjusted-R^2^
0.5 kHz	4.04 + 0.91(SRT) − 0.05(age) − 1.18(tinnitus) + 1.07(SAH)	0.8225	3.84 + 0.92(SRT) − 0.05(age)	0.8211
1 kHz	4.59 + 0.93(SRT)– 0.00(age)– 1.14(tinnitus)– 1.41(HT)	0.8881	2.89 + 0.94(SRT) − 0.00(age)	0.8852
2 kHz	5.23 + 0.90(SRT) + 0.6(age)– 0.59(tinnitus)– 0.89(HT)	0.7412	4.22 + 0.90(SRT) + 0.06(age)	0.741
4 kHz	18.41 + 0.68(SRT) + 0.15(age)	0.4109	18.41 + 0.68(SRT) + 0.15(age)	0.4109

**Fig. 2:**
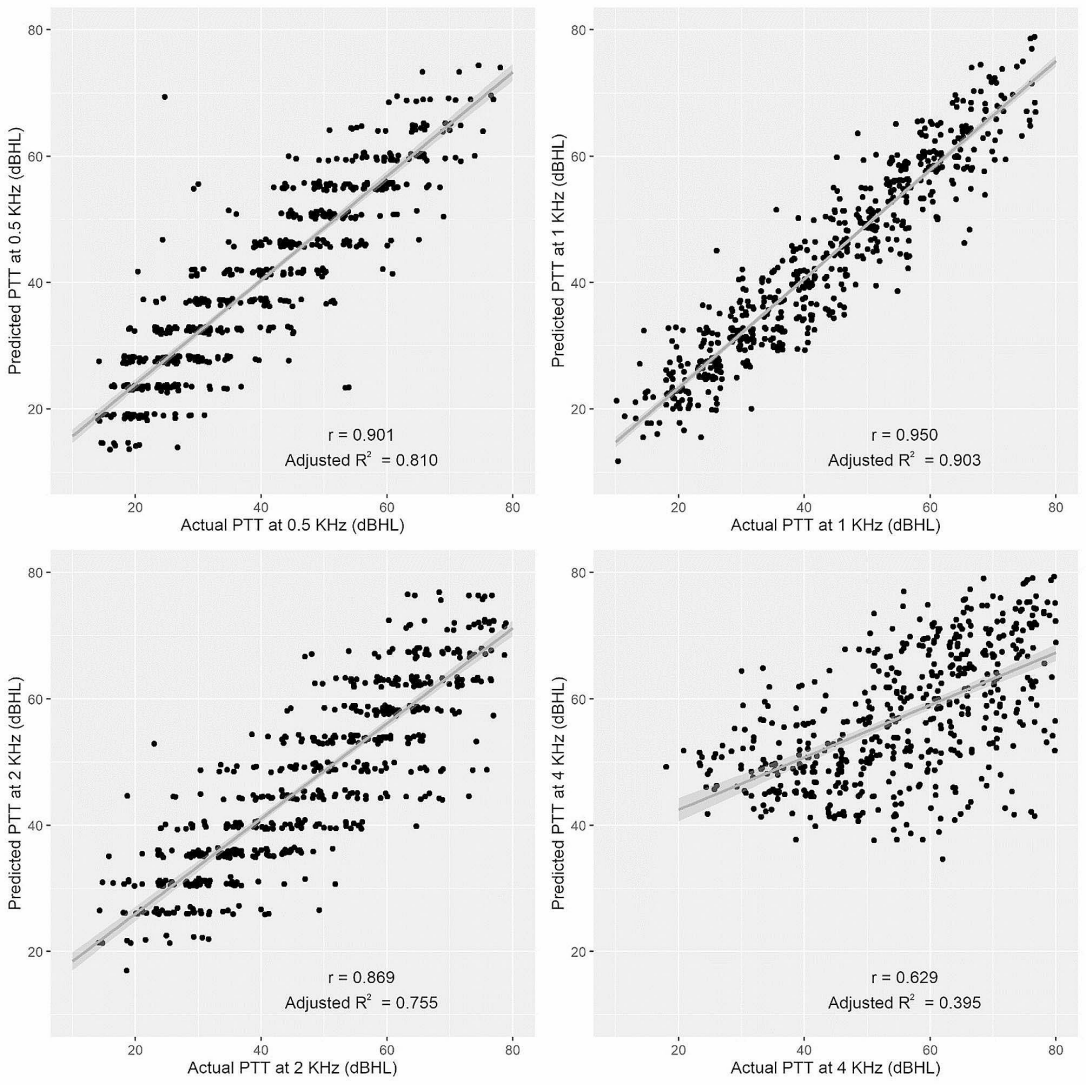
Pearson’s correlation coefficient describing the relationship between the actual and predicted pure-tone threshold (PTT) at 0.5, 1, 2, and 4 kHz among testing set

The prediction formula for PTTs using SRT and age from the training set showed a significantly high correlation between the actual and predicted values in the testing set, whose correlation plots are presented in Fig. 2. The adjusted-R^2^ of SRT and age in prediction of PTTs at 0.5, 1 and 2 kHz was high (greater than 75%). It indicates that SRT and age highly explained variability of PTTs. The adjusted-R^2^ of SRT and age with PTT at 4 kHz was low at 39%.

## Discussion

In our study, the SRT was the most dominant parameter used for the prediction of PTTs at each specific frequency across 0.5–4 kHz, after adjusting for age among elderly individuals with hearing loss using training and testing validation. The data was divided into those sets based on the concept that the training data was used to create the predictive formulas, whereas the testing data was used to provide an unbiased evaluation. We considered the age of patients for predicting the PTT, in addition to the SRT, which was first found to be a significant indicator of PTT by Alberti et al. in 1978 [41]. The prediction formula comprising SRT and age showed a highly positive correlation between the predicted and actual PTT at 0.5, 1, and 2 kHz, whereas a moderately positive correlation was found at 4 kHz. The variation of PTT prediction at 4 kHz could be explained by the effect of cochlear deterioration in the geriatric population, which primarily affected high-frequency regions. A typical audiogram of the geriatric population showed a flat configuration at the main speech frequencies, which were related to PTTs at 0.5, 1 and 2 kHz, and a sharp slope with elevated hearing thresholds at frequencies above 2 kHz [26, 28, 42–44]. Moreover, the progression of hearing loss in elderly individuals significantly worsens at high frequencies with advancing age. Studies have reported that the hearing threshold at high frequencies showed a higher rate of decline at 1.35 dB HL/year, which was only 0.29 dB HL/year for low frequencies [44]. Even though the deviation of PTT at 4 kHz was influenced by age and was responsible for the inconsistency in the hearing thresholds and variation in the dataset, the findings of our study indicate a moderate correlation between the predicted and actual PTTs at 4 kHz, which can be used clinically to facilitate early diagnosis or hearing aid fitting. However, the effect of cochlear deterioration in the geriatric population affecting high-frequency regions is not necessarily the only cause of the variation of PTT prediction. Another potential cause, particularly an age-related deficit in the central auditory pathway, is also a contributing factor for the variation of PTT prediction. Specifically, SRT obtained from patients with the age-related deficit in the central auditory pathways does not necessarily correlate with PTTs. In fact, the age-related deficit in the central auditory pathway can impair a number of speech test measures, including SRT [44]. As a result, SRT would be worse than expected and uncorrelated with PTTs.

The SRT has been considered as a significant indicator of the accuracy of the measured PTT [41]. In order to create the prediction formula for the PTT, SRT and age were generated from the training set, which showed a significantly high correlation between the actual and predicted values in the testing set and indicated the highly explained variability of the PTTs. The use of our formula to predict the PTTs has three advantages: to estimate the hearing threshold to establish a baseline for hearing amplification, to raise early awareness of an exaggerated hearing response, and to increase the efficiency of hearing examination in lack of healthcare providers.

First, the prediction formula can be practically applied to routine hearing evaluation to guide the prospective PTT at each specific frequency. SRT can be obtained to assess an overview of hearing thresholds during a testing session before performing pure tone audiometry. The increase in the severity of hearing loss and rising prevalence in elderly individuals necessitate early detection and intervention. Standard audiometry entails subjective actions, such as the correct response, and requires elderly individuals to attentively listen to unfamiliar sounds [2, 45, 46]. In addition, the geriatric population generally presents with complex health conditions, which can be an obstacle in the hearing examination. Studies have shown that the age-related changes in physical and cognitive function affect the consistency and accuracy of the results of audiometry [36–38, 47]. Moreover, the deterioration in intelligence and tinnitus cause listening difficulties, affecting the sensitivity of the PTT, which consequently increases the time for hearing evaluation, since it has to be repeated [48]. The PTT prediction formula can be used to assist audiologists to estimate the possible PTTs, thus obtaining an audiogram with efficiency. For example, when the patient has difficulty in responding to pure-tone stimuli, and he or she can only recognize the minimum hearing level for speech at 45 dB HL, based on our prediction model the predicted PTTs at 0.5, 1, 2 and 4 kHz will be estimated for 41.7, 45.2, 48.9 and 59.5 dB HL, respectively. If the patient responds to stimuli that are louder than the predicted PTTs, the audiologist is aware of possible exaggerated responses and reiterate the instructions at an early stage of testing to determine whether or not the responses represent the actual thresholds.

Second, the predicted PTTs could be used as a baseline for the prescription of hearing aid amplification in case of incomplete audiometry. Basically, the primary intervention to enhance hearing is a hearing aid, which requires a PTT at each specific frequency as a baseline to tune the appropriate amplification of the aid. The predicted PTTs are more likely to provide a hearing threshold that approximates the accurate value, which can help identify auditory dysfunction. An exaggerated pure-tone response can result in overamplification; thus, our formula can help avoid the risk of gain overamplification during hearing aid tuning. Thus, the PTTs obtained from the prediction formula can be useful in hearing aid fitting for early rehabilitation, especially in patients who cannot complete pure-tone audiometry.

Finally, the predicted PTT can increase the efficiency of hearing examination in lack of healthcare providers. The lack of hearing healthcare providers in many low- and middle-income countries is challenging, which may be the leading cause of limited access to hearing evaluation and rehabilitation centers [49]. Using the predicted PTTs can boost the confidence of less-experienced audiologists when confirming the possible PTTs. The audiologists can, at the very least, save more time with confidence during a testing session. Consequently, saving more time in each testing session can, in turn, provide efficient hearing assessment and eventually offer additional time for the audiologists to evaluate more patients. Furthermore, travel expenses present another hurdle to healthcare access [50–54]. Any hearing evaluation and rehabilitation clinics can apply the formulas to acquire predicted PTTs for elderly patients who cannot complete pure tone audiometry during an initial hearing evaluation rather than referring them straight away to other secondary centers with considerable expenses.

This study had some limitations. First, the model only provided information about air-conduction PTTs, which were computed from sensorineural hearing loss data. Therefore, our predictive model cannot be used in patients with mixed or conductive hearing loss. Even though our predictive formulas cannot be used to determine the type and configuration of hearing loss, the degree of hearing loss could be accessed by the average of predictive PTTs. Second, we used the most recent SRT and PTT, which were analyzed cross-sectionally, rather than longitudinally, to determine the variation within the same study population. Therefore, predictive PTTs used to compare with actual PTTs collected from this retrospective data with only one visit might not represent the actual PTTs obtained from prospective data at the subsequent visit. Thus, further research with a prospective study, instead of a retrospective study, is helpful to confirm the performance of the predictive model. Third, internal validation was performed using training and testing sets from the same Thai population with speech materials from Thai tonal language. As a result, the generalizability of the findings is solely limited to Thai language. Validation in a group of other tonal languages is required for further research to assess the performance of the predictive model. Fourth, as SRT obtained from patients with the age-related deficit in the central auditory pathway does not necessarily correlate with PTTs, the predicted PTTs obtained from these patients can also be affected. This limitation could, in turn, potentially affect the PTT prediction related to the suitability of hearing aid fitting. Finally, this study used data from the participants who completed audiometry. To apply this prediction formula, a prospective study should be conducted to measure hearing levels among people with hearing problems who are unable to complete pure-tone audiometry at the first visit. Thereafter, a prospective study should be conducted to illustrate the predictive performance of the PTT formula by comparing the predicted value at first visit and the actual PTT collected from the completed audiogram collected from multiple visits.

In summary, the prediction of PTTs using the SRT and age variables showed significantly high correlations between the actual and predicted values. It indicated high correlation of PTTs at 0.5, 1, and 2 kHz and moderate correlation of the SRT and age variables for predicting the PTT at 4 kHz. This formula can also be used during hearing examinations and interventions, especially in settings with a limited number of experienced audiologists.

## Data Availability

The datasets used and/or analyzed during the current study are available from the corresponding author on reasonable request.

## References

[1] Sliwinska-kowalska M. Chapter 19 - Hearing. In: Lotti M, Bleecker ML, editors. Handbook of Clinical Neurology [Internet]. Elsevier; 2015 [cited 2021 Dec 1]. pp. 341–63. (Occupational Neurology; vol. 131). https://www.sciencedirect.com/science/article/pii/B9780444626271000184. 10.1016/B978-0-444-62627-1.00018-4 26563797

[2] Musiek FE, Shinn J, Chermak GD, Bamiou DE (2017). Perspectives on the Pure-Tone Audiogram. J Am Acad Audiol.

[3] Davies RA. Chapter 11 - Audiometry and other hearing tests. In: Furman JM, Lempert T, editors. Handbook of Clinical Neurology [Internet]. Elsevier; 2016 [cited 2021 Dec 6]. pp. 157–76. (Neuro-Otology; vol. 137). https://www.sciencedirect.com/science/article/pii/B978044463437500011X. 10.1016/B978-0-444-63437-5.00011-X 27638069

[4] World Health Organisation. World Health Organisation Grades of hearing impairment [Internet]. 2008. https://ec.europa.eu/health/ph_risk/committees/04_scenihr/docs/scenihr_o_018.pdf.

[5] Said EA (2017). Health-related quality of life in elderly hearing aid users vs. non-users. Egypt J Ear Nose Throat Allied Sci.

[6] Ribeiro UASL, Souza VC, Lemos SMA (2019). Quality of life and social determinants in individual hearing AIDS users. CoDAS.

[7] World Health Organization. World report on hearing [Internet]. Geneva: World Health Organization. 2021 [cited 2022 Feb 23]. https://apps.who.int/iris/handle/10665/339913.

[8] Dindamrongkul R, Riewpaiboon W, Yimtae K, Krityakiarana W, Niyomphol W. Factors influencing making a choice and accessing a hearing aid among elders with hearing disability: mixed methods. International Journal of Human Rights in Healthcare [Internet]. 2022 Jan 1 [cited 2022 Jul 6];ahead-of-print(ahead-of-print). 10.1108/IJHRH-04-2022-0028.

[9] Magnuson A, Sattar S, Nightingale G, Saracino R, Skonecki E, Trevino KM (2019). A practical guide to geriatric syndromes in older adults with Cancer: a Focus on Falls, Cognition, Polypharmacy, and Depression. Am Soc Clin Oncol Educational Book.

[10] Inouye SK, Studenski S, Tinetti ME, Kuchel GA (2007). Geriatric syndromes: clinical, Research and Policy implications of a Core Geriatric Concept. J Am Geriatr Soc.

[11] Speros C. More Than Words: Promoting Health Literacy in Older Adults. undefined [Internet]. 2009 [cited 2021 Nov 30]; https://ojin.nursingworld.org/MainMenuCategories/ANAMarketplace/ANAPeriodicals/OJIN/TableofContents/Vol142009/No3Sept09/Health-Literacy-in-Older-Adults.aspx.

[12] Tenório GA, Ferrite S (2007). Estimativa do diferencial entre os Limiares Auditivos Subjetivos E Eletrofisiológicos em Adultos Normouvintes. Intl Arch Otorhinolaryngol.

[13] Walker JJ, Cleveland LM, Davis JL, Seales JS (2013). Audiometry screening and interpretation. Am Fam Physician.

[14] Lentz JJ, Walker MA, Short CE, Skinner KG (2017). Audiometric testing with pulsed, steady, and Warble tones in listeners with Tinnitus and hearing loss. Am J Audiol.

[15] Sidiras C, Sanchez-Lopez R, Pedersen ER, Sørensen CB, Nielsen J, Schmidt JH. User-Operated Audiometry Project (UAud)– Introducing an Automated User-Operated System for Audiometric Testing Into Everyday Clinic Practice. Frontiers in Digital Health [Internet]. 2021 [cited 2023 Oct 20];3. https://www.frontiersin.org/articles/10.3389/fdgth.2021.724748. 10.3389/fdgth.2021.724748 PMC8529271 34713194

[16] Oosterloo BC, Homans NC, Baatenburg de Jong RJ, Ikram MA, Nagtegaal AP, Goedegebure A (2020). Assessing hearing loss in older adults with a single question and person characteristics; comparison with pure tone audiometry in the Rotterdam Study. PLoS ONE.

[17] British Society of Audiology. Pure tone air and bone conduction threshold audiometry with and without masking [Internet]. British Society of Audiology. 2018 [cited 2023 May 28]. https://www.thebsa.org.uk/resources/pure-tone-air-bone-conduction-threshold-audiometry-without-masking/.

[18] Macrae JH (1991). Prediction of deterioration in hearing due to hearing Aid Use. J Speech Lang Hear Res.

[19] Roberts C (1970). Can hearing aids damage hearing?. Acta Otolaryngol.

[20] Markides A (1971). Do hearing aids damage the user’s residual hearing? (a literature survey). Br J Audiol.

[21] Thrailkill KM, Brennan MA, Jesteadt W (2019). Effects of amplification and hearing-aid experience on the contribution of specific frequency bands to loudness. Ear Hear.

[22] Determining Threshold Level for Speech [Internet]. American Speech-Language-Hearing Association. American Speech-Language-Hearing Association; 1988 [cited 2023 Oct 24]. https://www.asha.org/policy/gl1988-00008/.

[23] Newman JL. Development of Psychometrically Equivalent Speech Recognition Threshold Materials for Native Speakers of Samoan [Internet]. 2010. https://scholarsarchive.byu.edu/etd/2214.

[24] McMahon CM, Gopinath B, Schneider J, Reath J, Hickson L, Leeder SR, Mitchell P, Cowan R (2013). The need for Improved Detection and Management of Adult-Onset hearing loss in Australia. Int J Otolaryngol.

[25] Tsakiropoulou E, Konstantinidis I, Vital I, Konstantinidou S, Kotsani A (2007). Hearing aids: quality of life and socio-economic aspects. Hippokratia.

[26] Gelfand SA (2016). Essentials of Audiology.

[27] Stach BA (2010). Clinical audiology: an introduction.

[28] Katz J, Chasin M, English K, Hood LJ, Tillery KL. Handbook of Clinical Audiology [Internet]. 7th ed. Philadelphia: Wolters Kluwer Health; 2015 [cited 2021 Dec 10]. https://slh.lwwhealthlibrary.com/book.aspx?bookid=1174.

[29] dos Anjos WT, Ludimila L, de Resende LM, Costa-Guarisco LP (2014). Correlation between the hearing loss classifications and speech recognition. Rev CEFAC.

[30] Graham JT (1960). Evaluation of methods for Predicting Speech reception threshold. Arch Otolaryngol.

[31] Kim JM, Na MS, Jung KH, Lee SH, Han JS, Lee OH, Park SY (2016). The best-matched pure Tone Average and Speech Recognition threshold for different audiometric configurations. Korean J Otorhinolaryngol-Head Neck Surg.

[32] Ristovska L, Jachova Z, Kovacevic J, Radovanovic V, Hasanbegovic H (2021). Correlation between pure tone thresholds and speech thresholds. J Hum Res Rehabilitation.

[33] Chien CH, Tu TY, Shiao AS, Chien SF, Wang YF, Li ACI, Yang MJ (2008). Prediction of the pure-tone average from the speech reception and auditory brainstem response thresholds in a geriatric population. ORL J Otorhinolaryngol Relat Spec.

[34] Meister H, Schreitmüller S, Grugel L, Beutner D, Walger M, Meister I (2013). Examining speech perception in noise and cognitive functions in the elderly. Am J Audiol.

[35] Zekveld AA, Kramer SE, Festen JM (2011). Cognitive load during speech perception in noise: the influence of age, hearing loss, and cognition on the pupil response. Ear Hear.

[36] della Volpe A, Ippolito V, Roccamatisi D, Garofalo S, De Lucia A, Gambacorta V, Longari F, Ricci G, Di Stadio A (2020). Does unilateral hearing loss impair working memory? An Italian clinical study comparing patients with and without hearing aids. Front NeuroSci.

[37] Rosemann S, Thiel CM (2020). Neural signatures of Working Memory in Age-related hearing loss. Neuroscience.

[38] Kim S, Choi I, Schwalje AT, Kim K, Lee JH (2020). Auditory Working Memory Explains Variance in Speech Recognition in older listeners under adverse listening conditions. CIA.

[39] Chaiklin JB, Ventry IM, SPONDEE THRESHOLD MEASUREMENT (1964). A COMPARISON OF 2- AND 5-DB METHODS. J Speech Hear Disord.

[40] Amatyakul P, Audiology. Bangkok: Faculty of Medicine Ramathibodi Hospital; 1973.

[41] Alberti PW, Morgan PP, Czuba I (1978). Speech pure tone audiometry as a screen for exaggerated hearing loss in industrial claims. Acta Otolaryngol.

[42] Roup CM, Noe CM (2009). Hearing aid outcomes for listeners with high-frequency hearing loss. Am J Audiol.

[43] Profant O, Jilek M, Bures Z, Vencovsky V, Kucharova D, Svobodova V, Korynta J, Syka J (2019). Functional age-related changes within the human auditory system studied by Audiometric Examination. Front Aging Neurosci.

[44] Rigters SC, van der Schroeff MP, Papageorgiou G, Baatenburg de Jong RJ, Goedegebure A (2018). Progression of hearing loss in the Aging Population: repeated auditory measurements in the Rotterdam Study. AUD.

[45] Baguley DM, Cope TE, McFerran DJ. Chapter 32 - Functional auditory disorders. In: Hallett M, Stone J, Carson A, editors. Handbook of Clinical Neurology [Internet]. Elsevier; 2016 [cited 2021 Dec 1]. pp. 367–78. (Functional Neurologic Disorders; vol. 139). https://www.sciencedirect.com/science/article/pii/B9780128017722000321. 10.1016/B978-0-12-801772-2.00032-1 27719856

[46] Schlittenlacher J, Ellermeier W, Avci G (2017). Simple reaction time for broadband sounds compared to pure tones. Atten Percept Psychophys.

[47] Arehart KH, Souza P, Baca R, Kates JM (2013). Working memory, age and hearing loss: susceptibility to hearing aid distortion. Ear Hear.

[48] Chien CH, Tu TY, Chien SF, Li ACI, Yang MJ, Shiao AS, Wang YF (2006). Relationship between Mandarin Speech reception thresholds and pure-tone thresholds in the Geriatric Population. J Formos Med Assoc.

[49] World Health Organization. Multi-country assessment of national capacity to provide hearing care [Internet]. Geneva: World Health Organization. 2013 [cited 2023 Jan 26]. 47 p. https://apps.who.int/iris/handle/10665/339286.

[50] Valete-Rosalino CM, Rozenfeld S (2015). Auditory screening in the elderly: comparison between self-report and audiometry. Braz J Otorhinolaryngol.

[51] Yang TH, Chu YC, Chen YF, Chen MY, Cheng YF, Wu CS, Huang HM (2021). Diagnostic validity of self-reported hearing loss in Elderly Taiwanese individuals: diagnostic performance of a hearing Self-Assessment Questionnaire on Audiometry. Int J Environ Res Public Health.

[52] Kovalová M, Mrázková E, Škerková M, Čada Z, Janoutová J (2021). The importance of screening for hearing loss in the Elderly. Otolaryngol Pol.

[53] Kornelsen J, Khowaja AR, Av-Gay G, Sullivan E, Parajulee A, Dunnebacke M, Egan D, Balas M, Williamson P (2021). The rural tax: comprehensive out-of-pocket costs associated with patient travel in British Columbia. BMC Health Serv Res.

[54] Yong M, Willink A, McMahon C, McPherson B, Nieman CL, Reed NS, Lin FR (2019). Access to adults’ hearing aids: policies and technologies used in eight countries. Bull World Health Organ.

